# The viscosity and processing of molten lunar regolith

**DOI:** 10.1038/s41598-025-87761-7

**Published:** 2025-01-31

**Authors:** James Bowen, Vibha Levin Prabhu, Sungwoo Lim, Mahesh Anand

**Affiliations:** 1 Faculty of Science, Technology, Engineering and Mathematics, The Open University, Walton Hall, Milton Keynes, MK7 6AA UK; 2 European Space Resources Innovation Centre, Luxembourg Institute of Science and Technology, Belvaux, L-4422 Luxembourg; 3 Surrey Space Centre, University of Surrey, Surrey, Guildford, GU2 7XH UK

**Keywords:** In situ resource utilisation, JSC-1A, Lunar construction, Lunar simulant, Regolith, Viscosity, rheology, Viscosity, Engineering, Materials science, Physics

## Abstract

Establishing a permanent, self-sufficient habitat for humans on planetary bodies is critical for successful space exploration. In-situ resource utilisation (ISRU) of locally available resources offers the possibility of an energy-efficient and cost-effective approach. This paper considers the high-temperature processing of molten lunar regolith under conditions which represent the lunar environment, namely low gravity, low temperature, and negligible atmospheric pressure. The rheological properties of the low-titanium lunar mare regolith simulant JSC-1A are measured using concentric cylinder rheometry and these results are used to explore the influence of viscosity on processing operations involving the flow of molten regolith for fabricating construction components on the Moon surface. These include the delivery of molten regolith within an extrusion-based 3D printing technique and the ingress of molten regolith into porous structures. The energy and power required to establish and maintain sufficiently high temperatures for the regolith to remain in the liquid state are also considered and discussed in the context of lunar construction.

**Supplementary Information:**

The online version contains supplementary material available at 10.1038/s41598-025-87761-7.

##  Introduction

Establishing a permanent, self-sufficient habitat for humans on planetary bodies including the Moon and Mars is critical to the success of space exploration missions. Essential items for developing extra-terrestrial habitats require diverse ranges of materials capable of supporting applications including power generation, life support, and structural engineering. In-situ resource utilisation (ISRU) of geological materials available locally presents an attractive, efficient method for habitat construction, in terms of cost, logistics, and energy when compared to transporting materials from Earth. The primary components of lunar regolith – in order of decreasing abundance – are glass, plagioclase, olivine, pyroxene, and ilmenite. The concentration of each component is location-dependent on the lunar surface^1^. In order to study the processing of both lunar and Martian regolith a series of more than thirty soil simulants have been established^2–9^. These are multi-component powders, whose thermal^10,11^, optical^12,13^, and electromagnetic^14,15^ properties have been studied.

Research which has considered the processing of lunar simulants has tended to focus on physical properties including their shape^16^, handling^17^ and flowability^18,19^. The suitability of these materials for manufacturing low-porosity structures capable of supporting extraterrestrial construction has also been explored^20–23^, with the properties of the processed simulants after mechanical compaction^24,25^ and 3D printing^26,27^ also receiving attention. Heating the simulants to temperatures at which sintering and melting occur^28,29^ leads to the formation of crystalline phases embedded within ceramics and glasses, the physicochemical properties of which have also been studied^30,31^. The microstructure of the resultant material is also an area of investigation^21,23,32,33^, as this determines the usefulness of the material for construction purposes. The possibility of extracting life-supporting resources, including oxygen, water and iron, from the regolith, when heated to high temperatures, is a topic of great interest^34–36^. Resource extraction is also possible using reduction^37^, electrolysis, or pyrolysis^38^.

Although research which seeks to address the feasibility of producing commodities from lunar and Martian regolith is underway, few studies have considered the rheological properties of the lunar and Martian simulants once heated to the molten state. Noteworthy investigations include a study into the behaviour of Mars soil simulant JSC-Mars-1 when mixed with water^39^, a comparison of three lunar simulants which considered their crystallisation behaviour when supercooled^40^, and the implications of the Marangoni effect for processing the molten liquid^41^. At volume fractions in the range 0.39–0.49, the aqueous JSC-Mars-1 slurry exhibited viscoelasticity, behaving like an elastic solid at short timescales, and like a yield stress fluid at longer timescales. The lunar simulants JSC-1A, Stillwater norite, and Stillwater anorthosite exhibited Newtonian behaviour; crystallisation occurred rapidly once the norite and anorthosite were at temperatures lower than their liquidus (≈ 1450 °C and ≈ 1550 °C respectively), though JSC-1A did not begin to crystallise until ≈ 1200 °C^41^. The liquidus of JSC-1A is ≈ 1325 °C, representing the temperature above which the material is completely liquid, and the maximum temperature at which crystals can co-exist with the melt in thermodynamic equilibrium.

This paper considers the temperature-dependent rheology of molten lunar soil simulant JSC-1A, hereafter referred to as lunar soil simulant (LSS), at temperatures in the range 1200–1600 °C, and the implications for its processing and handling for the purpose of manufacturing commodities under lunar gravity and at the temperatures and atmospheric pressures encountered in the lunar environment. The JSC-1A LSS approximates a low-titanium mare regolith in its composition^42^ and is commonly employed for ISRU studies associated with materials derived from the lunar surface. In this work, the dynamic viscosity of the melt is measured using a concentric cylinder furnace rheometer, and an analytical equation is generated which expresses the temperature-dependent viscosity. The implications of these results for lunar processing are explored in an engineering context, considering the effect of temperature on melt flow through conduits, the ingress of molten liquid into porous structures, and the rapidity with which the melt viscosity could change upon exposure to the lunar environment. The results support the determination of optimal parameters for developing a material delivery mechanism of an extrusion-based 3D printing technique, suitable for fabricating lunar habitats/infrastructure. This is a preliminary study with limited applicability to real lunar conditions, yet the insights generated will be useful for planning regolith handling operations and their energy requirements.

## Materials and methods

###  Materials

JSC-1A is a lunar simulant that approximates a low-titanium mare regolith and contains glass (49%), major crystalline silicate phases of plagioclase (37%) and olivine (9%), as well as minor oxide phases of chromite (1.1%), albite (0.3%), and ilmenite (< 0.1%). It is mined from a volcanic ash deposit in a commercial cinder quarry located in the San Francisco volcano field near the Merriam Crater outside Flagstaff, Arizona. JSC-1A begins to melt in the temperature range 1125–1175 ^o^C with melting complete at temperatures > 1200 ^o^C^40,41,43^.

### Rheometry

The shear rate-dependent dynamic viscosity of molten JSC-1A was measured using an FRS 1800 furnace rheometer (Anton Paar, Austria). The measuring system was a graphite bob and cup, the diameters of which were 19 mm and 30 mm respectively. Approximately 50 g of JSC-1A powder was added to the cup at room temperature, followed by the installation of the cup inside the furnace rheometer. The bob was placed into contact with the top of the powder, maintaining a normal compressive load of ≈ 3 N. The system was then heated to a temperature of 1600 °C with the heating rate ≈ 25 °C min^-1^. The cylinder rheometer was able to penetrate the sample and move to the measurement position after the sample had melted.

During equilibration at 1600 °C, the JSC-1A melt was continuously sheared at high stress to remove any air bubbles which might have formed during the phase change. Shear rate sweeps were then performed at a series of 10 constant temperatures; the temperature range was 1600 °C to 1200 °C, with the system cooling naturally and equilibrating between each shear rate sweep. The shear rate range was 0.1–30 s^-1^ for all sweeps, with data discernible at shear rates > 0.5 s^-1^ at all temperatures, with data discernible at shear rates < 0.2 s^-1^ at temperatures < 1300 °C. All measurements were performed under an argon atmosphere, maintaining a gas flow rate of ≈ 1 L min^-1^ throughout the test sequence.

## Experimental results

### Effect of shear rate

**Fig. 1:**
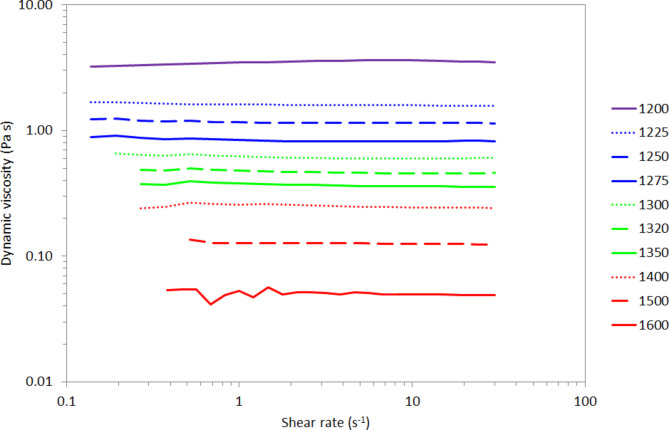
The dynamic viscosity of molten JSC-1A in the temperature range 1200–1600 °C does not depend on the shear rate. Measurements were performed in reverse order of temperature, i.e., highest temperature first.

Figure 1 shows the effect of shear rate on the dynamic viscosity of JSC-1A melt at temperatures in the range 1200 °C to 1600 °C. The melt exhibits Newtonian behaviour at all temperatures measured, i.e., The dynamic viscosity, $\:\eta\:$, is the constant of proportionality which relates the shear rate, $\:\dot{\gamma\:}$, to the shear stress, $\:\tau\:$, in the fluid according to Eq. 1:

1 $$\:\tau\:=\eta\:\dot{\gamma\:}$$

The mean dynamic viscosity of the melt at 1600 °C is 0.050 ± 0.001 Pa s, whereas at 1200 °C the mean dynamic viscosity is 3.481 ± 0.056 Pa s., i.e. The viscosity of molten JSC-1A LSS is approximately 70 times greater at 1200 °C compared to 1600 °C.

### Effect of temperature

**Fig. 2:**
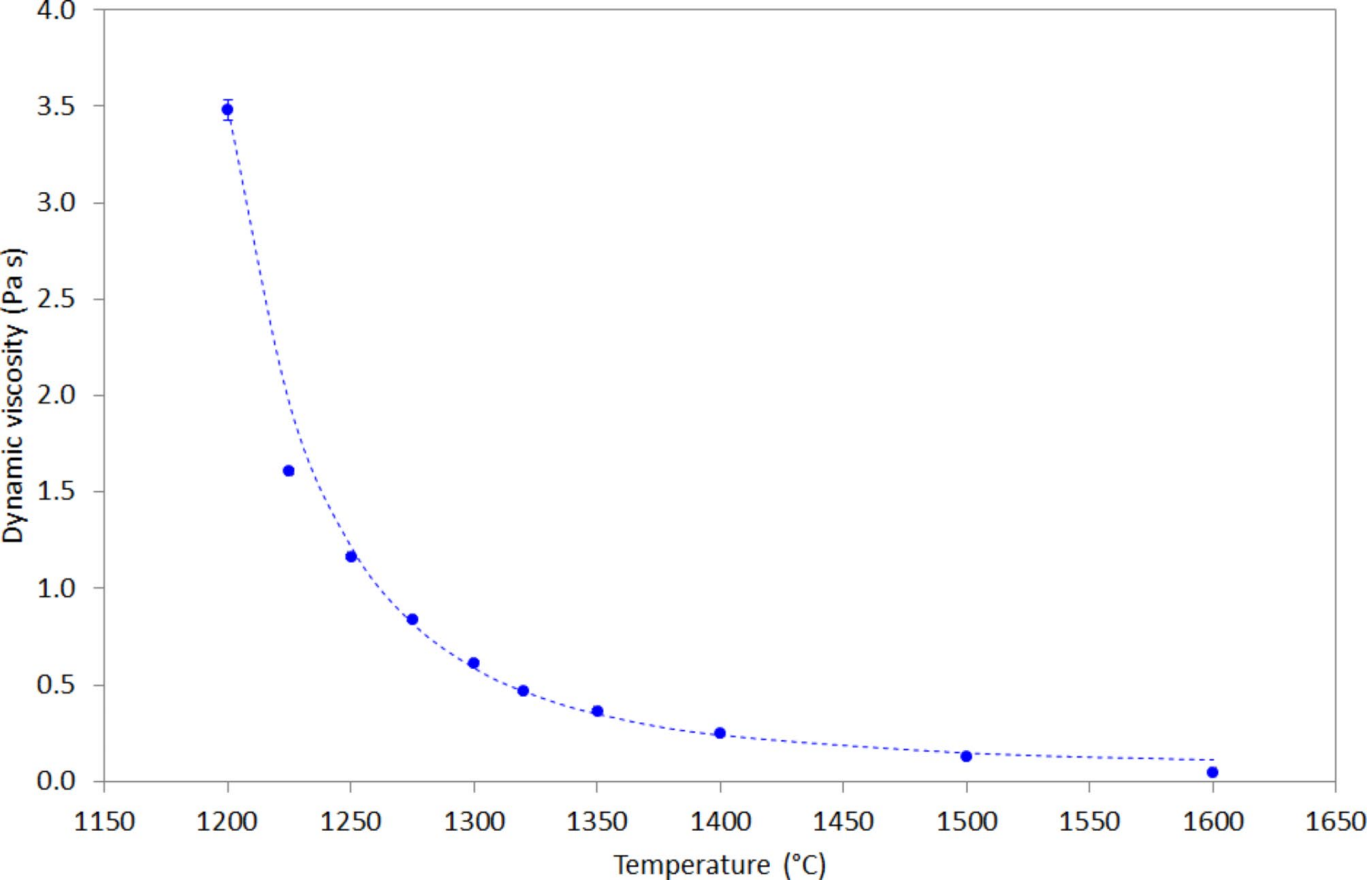
Effect of temperature on the dynamic viscosity of molten JSC-1A in the temperature range 1200-1600 °C; circles show measured data points; dashed line shows the theoretical relationship described by Eq. 2.

Figure 2 shows the effect of temperature on the dynamic viscosity of molten JSC-1A LSS at temperatures in the range 1200 °C to 1600 °C. The viscosity decreases approximately exponentially with increasing temperature. At $\:T=$ 1200 °C, the dynamic viscosity was measured as $\:\eta\:=$ 3.48 Pa s, whereas at $\:T=$ 1600 °C, the dynamic viscosity was measured as $\:\eta\:=$ 0.05 Pa s. The analytical expression shown in Eq. 2 was found to fit the data reasonably well (R^2^ = 0.989) using a least-mean-squares (LMS) algorithm:

2 $$\:\eta\:\left(T\right)=A{e}^{\left(\frac{B}{T-C}\right)}+D$$

where $\:\eta\:\left(T\right)$ is the temperature-dependent dynamic viscosity, $\:T$ is the temperature in Kelvin, $\:A$ and $\:D$ are constants with units Pa s, while $\:B$ and $\:C$ are constants with units K. The values of the constants are given in Table 1.

**Table 1:** Fitting constants for Eq. 2, which describes the temperature-dependent dynamic viscosity of the JSC-1A melt.

Parameter	Units	Value
$\:A$	Pa s	4.9785 × 10^− 3^
$\:B$	K	1.6514 × 10^3^
$\:C$	K	1.2205 × 10^3^
$\:D$	Pa s	5.000 × 10^− 2^

Two points of discrepancy between the measured viscosities and the theoretical values calculated using Eq. 2 are the data points at 1225 °C and 1600 ^o^C. We speculate that some crystallisation may be occurring within the melt at temperatures < 1250 °C. At temperatures below 1200 °C there was sufficient solid material forming that measurements had to be stopped in order to preserve the integrity of the instrument. However, Fig. 1 shows that the melt appears Newtonian over the shear rate range measured here, hence any crystallised material only accounts for a tiny volume fraction, otherwise non-Newtonian behaviour would be evident in the shear rate sweeps. At 1600 ^o^C, the theoretical value according to Eq. 2 (0.11 Pa s) deviates from the measured data (0.05 Pa s) by a significant amount. However, the parameters in Table 1 are the outcome of a robust LMS fitting and the implications of this deviation are discussed in § 4.4.

The four-parameter Eq. 1 yielded a reasonable fit of theory to experimental data (R^2^ = 0.989) whereas an Arrhenius-type equation^44^ yielded a poorer fit (R^2^ = 0.830). However, the activation energy associated with the Arrhenius fit was approximately 150 kJ mol^− 1^, which may be of benefit to researchers who seek material properties for their models and simulations.

## Theoretical interpretation and discussion

The processing of lunar regolith is likely to involve a variety of engineering unit operations which are typically employed in manufacturing and fabrication. The scale at which such operations might take place is still to be determined, though the use of regolith for habitat construction is widely anticipated. In order to demonstrate the impact of the temperature-dependent rheology of molten JSC-1A LSS, a number of scenarios will be considered:

- The energy required to heat regolith to the molten state.
- The power required to maintain regolith in the molten state.
- The flow of molten regolith through a conduit with circular cross-section.
- The ingress of melt into porous structures.

We assume that the rheology of the lunar regolith in the molten state will be comparable to the rheology of molten JSC-1A LSS because of their similar compositions. The continuous function shown in Eq. 2 is used for all calculations which require the temperature-dependent viscosity. Thermophysical properties of the lunar regolith, such as heat capacity, are used from the existing literature wherever they were found to be available.

### The energy required to heat regolith to the molten state

*How much energy is required to heat lunar regolith to its melting point?*

The energy, $\:E$, required to heat mass $\:m$ of material between two temperatures is given by Eq. 3:

3 $$\:E=m{C}_{p}{\Delta\:}T$$

where $\:{C}_{p}$ is the heat capacity of the material, and $\:{\Delta\:}T$ is the temperature difference.

The temperature-dependent heat capacity, $\:{C}_{p}\left(T\right)$, of solid lunar regolith is given by Eq. 4 ref^43^:

4 $$\:{C}_{p}\left(T\right)={c}_{1}+{c}_{2}T+{c}_{3}{T}^{-2}$$

where $\:{c}_{1}$, $\:{c}_{2}$, and $\:{c}_{3}$ are coefficients, and $\:T$ is the temperature in Kelvin. For solid low-titanium mare regolith the values of the constants are shown in Table 2.

**Table 2:** Fitting coefficients for Eq. 4, which describes the temperature-dependent heat capacity of solid regolith^43^.

Parameter	Units	Value
$\:{c}_{1}$	J kg^-1^ K^-1^	909.3
$\:{c}_{2}$	J kg^-1^ K^-2^	0.2870
$\:{c}_{3}$	J kg^-1^ K	-2.469 × 10^7^

The Lunar Reconnaissance Orbiter has mapped the temperature on the Moon since 2009, revealing that the daytime temperatures near the lunar equator reach 120° C whereas night-time temperatures reach − 130° C. The coldest temperature measured is around − 250° C, in the Hermite Crater and at the bottom of several permanently shadowed craters at the lunar south pole^45^. Assuming an ambient starting temperature of $\:{T}_{a}=$ 100 K, the energy required to heat 1.0 kg of solid lunar regolith to the molten state, i.e. 1200 °C (1473 K), is $\:{E}_{1}=\:$1.207 MJ. The latent heat of melting for low-titanium mare regolith is 457.7 kJ kg^-1^^43^; hence the additional energy required for the solid/liquid phase transition is $\:{E}_{2}=$ 0.4577 MJ. Therefore, the total energy required to heat 1.0 kg JSC-1A from 100 K to the molten state at 1200 °C (1473 K) is $\:{E}_{1}+{E}_{2}=\:$1.665 MJ.

Once molten, the heat capacity of low-titanium mare regolith is approximately constant at 1,539 J kg^-1^ K^-1^^43^. The energy required to heat molten lunar regolith (MLR) from 1200 °C to 1600 °C is therefore $\:{E}_{3}=\:$615.6 kJ. Therefore, the total energy required to heat 1.0 kg lunar regolith from 100 K to 1873 K is $\:{E}_{1}+{E}_{2}+{E}_{3}=\:$2.28 MJ. An energy of 1 MJ is equivalent to 0.2778 kWh, hence the specific energy can be expressed as 2.28 MJ kg^-1^ or 0.633 kWh kg^-1^, which could be useful when considering scale-up of regolith processing and associated unit operations.

### The power required to maintain lunar regolith in the molten state

There are various conceivable scenarios for handling MLR. As a basic calculation, we consider the situation where a mass of MLR is supported by a heated substrate which has a higher temperature than the MLR. The substrate is composed of a material such as niobium (m.p. 2477 °C), molybdenum (m.p. 2623 °C), or tungsten (m.p. 3422 °C). Conductive heat transfer occurs from the substrate into the MLR and radiative heat transfer occurs from the surface of the MLR. The heat (i.e. power) transferred into the MLR must balance the energy lost. In the lunar environment the convective loss is assumed to be negligible, and so radiation will be the dominant mechanism for energy loss from the MLR.

**Fig. 3:**
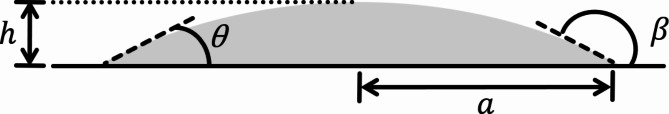
Droplet shape and associated parameters for molten regolith on a planar substrate.

Assuming a 1.0 kg droplet of MLR sits on the heated substrate (Fig. 3), the shape of the droplet is approximated by a spherical cap, the surface area, $\:S$, of which is given by Eq. 5:

5 $$\:S=\pi\:\left({a}^{2}+{h}^{2}\right)$$

where $\:a$ is the droplet radius and $\:h$ is the height of the droplet centre; $\:\theta\:$ and $\:\beta\:$ quantify the liquid wettability on the solid surface, where $\:\theta\:$ is termed the contact angle.

The temperature-dependent density, $\:\rho\:\left(T\right)$, of MLR is described by Eq. 6 ref^43^:

6 $$\:\rho\:\left(T\right)=\frac{{r}_{1}}{{r}_{2}+{r}_{3}\left(T-{T}_{0}\right)}$$

where $\:{r}_{1}$, $\:{r}_{2}$, and $\:{r}_{3}$ are coefficients, $\:T$ is the temperature in Kelvin, and $\:{T}_{0}$ is a reference temperature (also in Kelvin) which indicates the upper limit of validity for the expression. For low-titanium mare regolith the values of the coefficients are given in Table 3.

**Table 3:** Fitting constants for Eq. 6, which describes the temperature-dependent density of molten regolith^43^.

Parameter	Units	Value
$\:{r}_{1}$	kg m^-3^	63,480
$\:{r}_{2}$	-	23.01
$\:{r}_{3}$	K^-1^	1.612 × 10^− 3^

In the temperature range 1200 °C to 1600 °C, the density of MLR varies between 2,854 kg m^-3^ and 2,774 kg m^-3^. For a 1.0 kg MLR droplet, assuming a density of 2,814 kg m^-3^ intermediate between these two values, the droplet volume is 356 mL. The surface tension of molten JSC-1A is approximately 0.35 N m^-1^^41^ suggesting that the liquid will ‘wet’ chemically similar surfaces reasonably well. Assuming a contact angle $\:\theta\:=$ 10^o^ for a solid/liquid interface with good wetting, a 1.0 kg droplet (356 mL) exhibits radius $\:a=$ 60.2 mm, height $\:h=$ 50.5 mm, and surface area $\:S=$ 0.0194 m^2^. These calculations are performed using equations S1-S6 in the Supporting Information.

The temperature-dependent radiative power loss, $\:{P}_{rad}\left(T\right)$, can be estimated using the Stefan-Boltzmann law, Eq. 7:

7 $$\:{P}_{rad}\left(T\right)=S\epsilon\:\sigma\:\left({T}_{b}^{4}-{T}_{a}^{4}\right)$$

where $\:S$ is the surface area of the liquid/vapour interface, $\:\epsilon\:$ is the emissivity of the material, $\:\sigma\:$ is the Stefan-Boltzmann constant 5.6704 × 10^− 8^ W m^-2^ K^-4^, $\:{T}_{b}$ is the temperature of the material, and $\:{T}_{a}$ is the ambient temperature; both temperatures in Kelvin.

The emissivity of JSC-1A is assumed to be $\:\epsilon\:=$ 0.90^46^; according to Wien’s displacement law, Eq. 8, over the temperature range 1200–1600 °C the peak wavelengths, $\:{\lambda\:}_{peak}$, over which the emission intensity will be highest is 1.55–1.97 μm.

8 $$\:{\lambda\:}_{peak}=\frac{b}{{T}_{b}}$$

where $\:b$ is Wien’s displacement constant 2.8978 × 10^− 3^ m K.

Assuming the temperature of the 1.0 kg droplet of regolith is to be maintained at $\:{T}_{b}=$ 1600 °C, and the ambient temperature is $\:{T}_{a}=$ 100 K, the radiative power emitted from the droplet is $\:{P}_{rad}=$ 12.2 kW. However, $\:{P}_{rad}$ will decrease as the temperature of the regolith decreases; at $\:{T}_{b}=$ 1200 °C, $\:{P}_{rad}=$ 4.7 kW. These values, which represent the minimum power to be supplied by the heated substrate, could also be expressed per unit area of exposed MLR, i.e. $\:\frac{{P}_{rad}}{S}$; therefore at $\:{T}_{b}=$ 1600 °C, $\:\frac{{P}_{rad}}{S}=$ 628 kW m^-2^, whereas at $\:{T}_{b}=$ 1200 °C, $\:\frac{{P}_{rad}}{S}=$ 240 kW m^-2^.

Conserving energy in the lunar environment is an important consideration, and so the ‘holding temperature’ of materials while they are in-between unit operations can have a significant impact. If the substrate heating was switched off and the MLR were allowed to cool, the time over which the droplet temperature decreases from 1600 °C to 1200 °C, the onset of solidification, is $\:t=$ 84.6 s, calculated using Eq. 9:

9 $$\:t=\frac{m{C}_{p}{\Delta\:}T}{{P}_{rad}\left(T\right)}$$

**Fig. 4:**
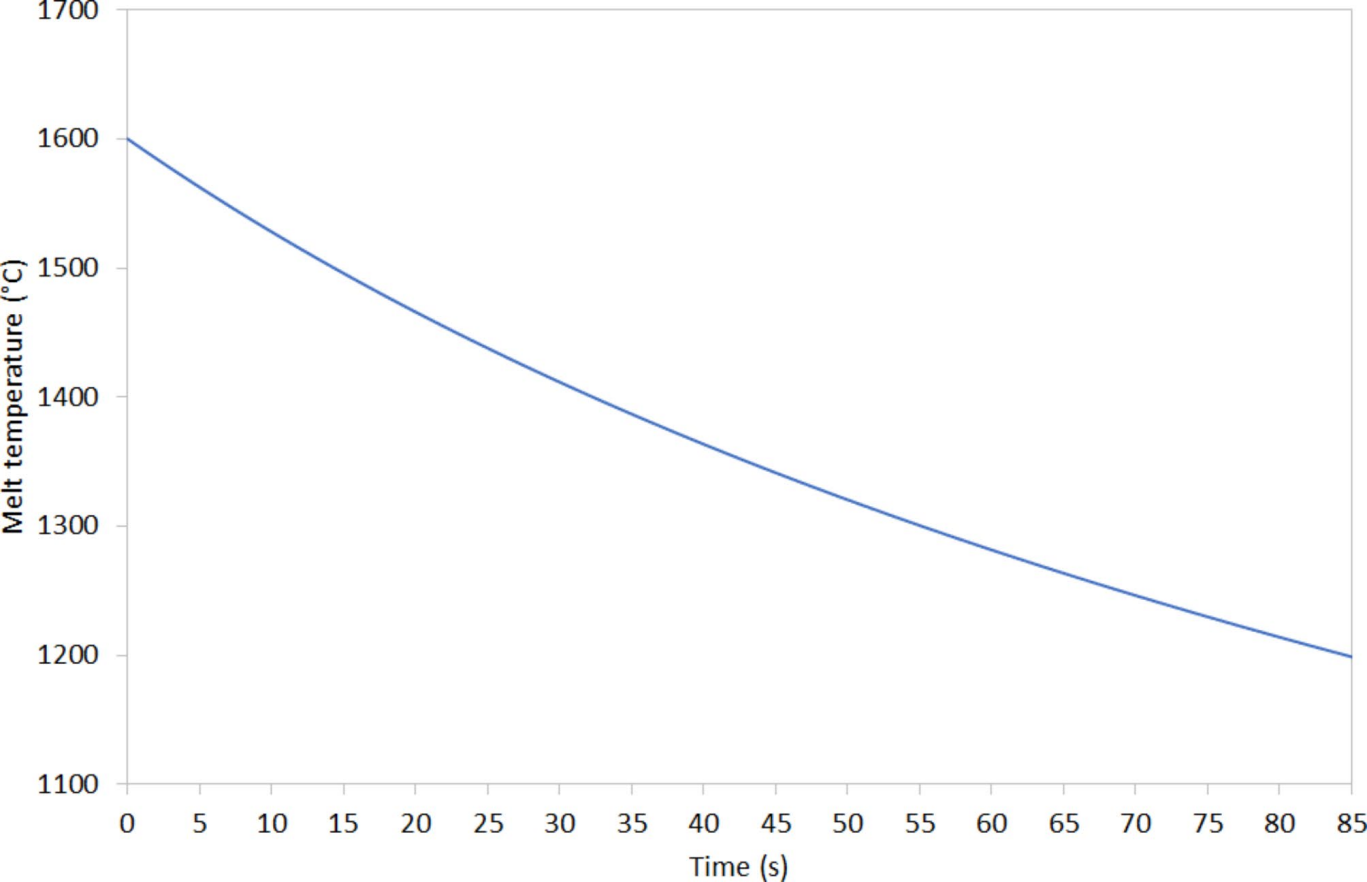
Effect of radiative loss on the temperature of molten lunar regolith at an ambient temperature of 100 K.

Note the non-linear decrease in $\:{P}_{rad}\left(T\right)$ as the temperature decreases, illustrated in Fig.4.

This result assumes that the droplet temperature is uniform throughout its volume, but how likely is this? The thermal diffusivity, $\:\alpha\:$, of a material is a measure of how rapidly heat is transferred through that material, and is given by:

10 $$\:\alpha\:=\frac{k}{\rho\:{C}_{p}}$$

where $\:k$ is the thermal conductivity of the material.

In the temperature range 1200 °C to 1600 °C, the thermal diffusivity of MLR varies between 0.020 mm^2^ s^-1^ to 0.007 mm^2^ s^-1^^41^. These are low values – for example, water at 25 ^o^C has a thermal diffusivity of 0.143 mm^2^ s^-1^. Therefore, it is likely that a temperature gradient will rapidly develop across the droplet, with the surface much cooler than the droplet interior.

**Fig. 5:**
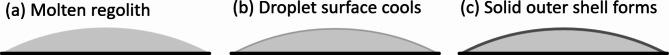
A core of molten regolith on a heated planar substrate, housed within a solid outer shell.

This rapid drop in the temperature of the MLR would lead to a pronounced increase in viscosity, closely followed by solidification at unheated surfaces and interfaces. It is likely that the outermost edge of the spherical cap would undergo solidification, leaving a molten core within a solid outer shell, as illustrated in Fig. 5. Given that the thermal conductivity of the solid regolith should be lower than the thermal conductivity of the molten regolith^43^, maintaining a large thermal gradient across the solid outer shell could be a worthwhile strategy for reducing the power required to maintain molten regolith at high temperature. The solidification of the outer surface, while the interior remains molten, is likely to occur for any shape of deposited or extruded molten regolith. The evolution of this phenomenon will depend on the size and mass involved, governed by an interplay of radiative cooling and heat conduction.

### The flow of molten regolith through a conduit with circular cross-section

*How does viscosity affect the flow of molten regolith?*

Local heating of solid regolith into a molten liquid, followed by gravity-driven flow, is one possible distribution mechanism for construction purposes. For example, extrusion-based additive manufacturing methods will require delivery of melt to a target location. In order to transport molten regolith as a liquid, it is unlikely that pumping or piston-based delivery will be suitable, given the high temperatures involved. How feasible is gravity-driven flow?

**Fig. 6:**
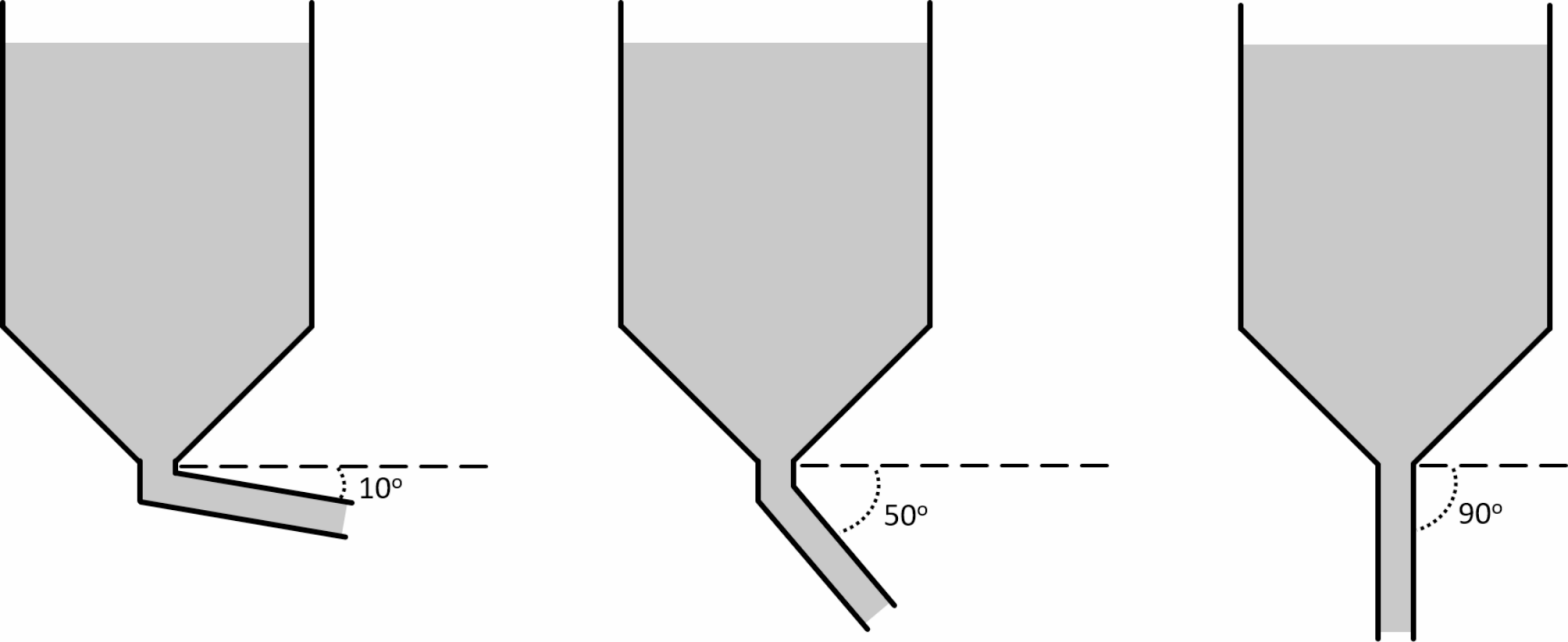
Apparatus for controlling the gravity-driven mass flow rate of molten regolith.

Equation 11 describes the temperature-dependent mass flow rate of molten regolith along a conduit with circular cross-section^47^:

11 $$\:\dot{m}\left(T\right)=\frac{\pi\:\rho\:\left(T\right){D}^{2}}{4}\left(u+\frac{\rho\:\left(T\right)g{D}^{2}\text{sin}\alpha\:}{32\eta\:\left(T\right)}\right)$$

where $\:\dot{m}\left(T\right)$ is the temperature-dependent mass flow rate, $\:\rho\:\left(T\right)$ is the temperature-dependent density, $\:D$ is the conduit diameter, $\:u$ is the flow velocity, $\:g$ is the acceleration due to gravity, and $\:\alpha\:$ is the tilt angle from the horizontal (Fig. 6).

**Fig. 7:**
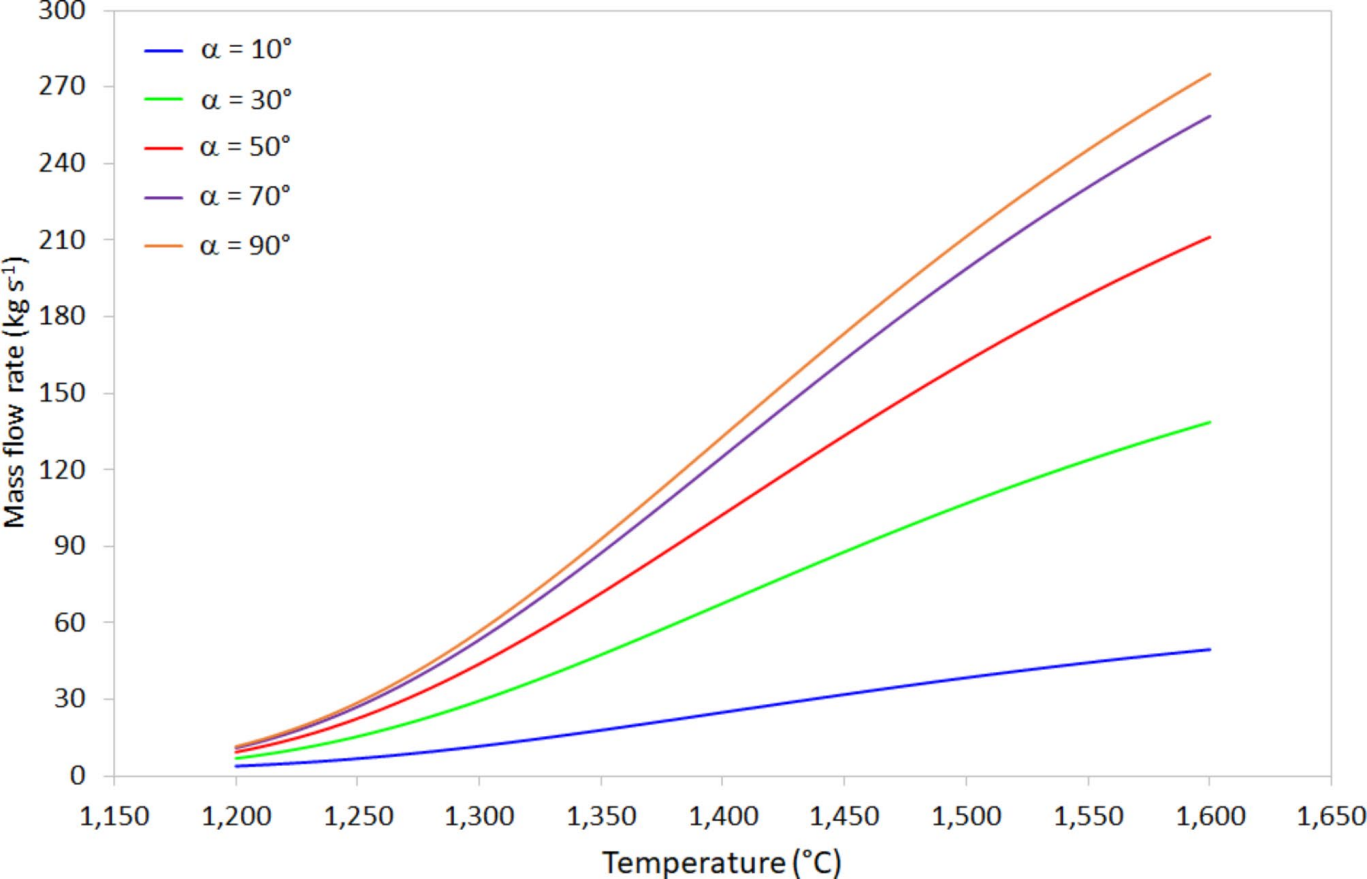
Effect of temperature on the mass flow rate of molten regolith in the temperature range 1200-1600 °C for conduit tilt angles, α, of 10°, 30°, 50°, 70°, and 90° from the horizontal (see Fig. 6 for schematic).

The mass flow rate is inversely proportional to the viscosity, depending strongly on both conduit diameter and tilt angle. For the example shown in Fig. 7, using the value of gravitational acceleration on the Moon (1.625 m s^-2^), the conduit internal diameter is 0.10 m, the velocity is 0.10 m s^-1^, and the tilt angle varies between 10^o^ and 90^o^ downwards from the surface horizontal. At $\:T=$ 1200 °C and 10^o^ angle, the mass flow rate $\:\dot{m}=$ 3.86 kg s^-1^, whereas at $\:T=$ 1600 °C and 10^o^ angle the mass flow rate $\:\dot{m}=$ 49.6 kg s^-1^. Similarly, at $\:T=$ 1200 °C and 90^o^ angle, the mass flow rate $\:\dot{m}=$ 11.6 kg s^-1^, whereas at $\:T=$ 1600 °C and 90^o^ angle the mass flow rate $\:\dot{m}=$ 275.0 kg s^-1^. These represent variations greater than an order-of-magnitude, which means the design of any system involving the flow of MLR, such as an additive manufacturing process, should carefully consider the implications of failing to maintain a constant melt temperature.

### The power/energy required to achieve a specified mass flow rate of molten lunar regolith

**Fig. 8:**
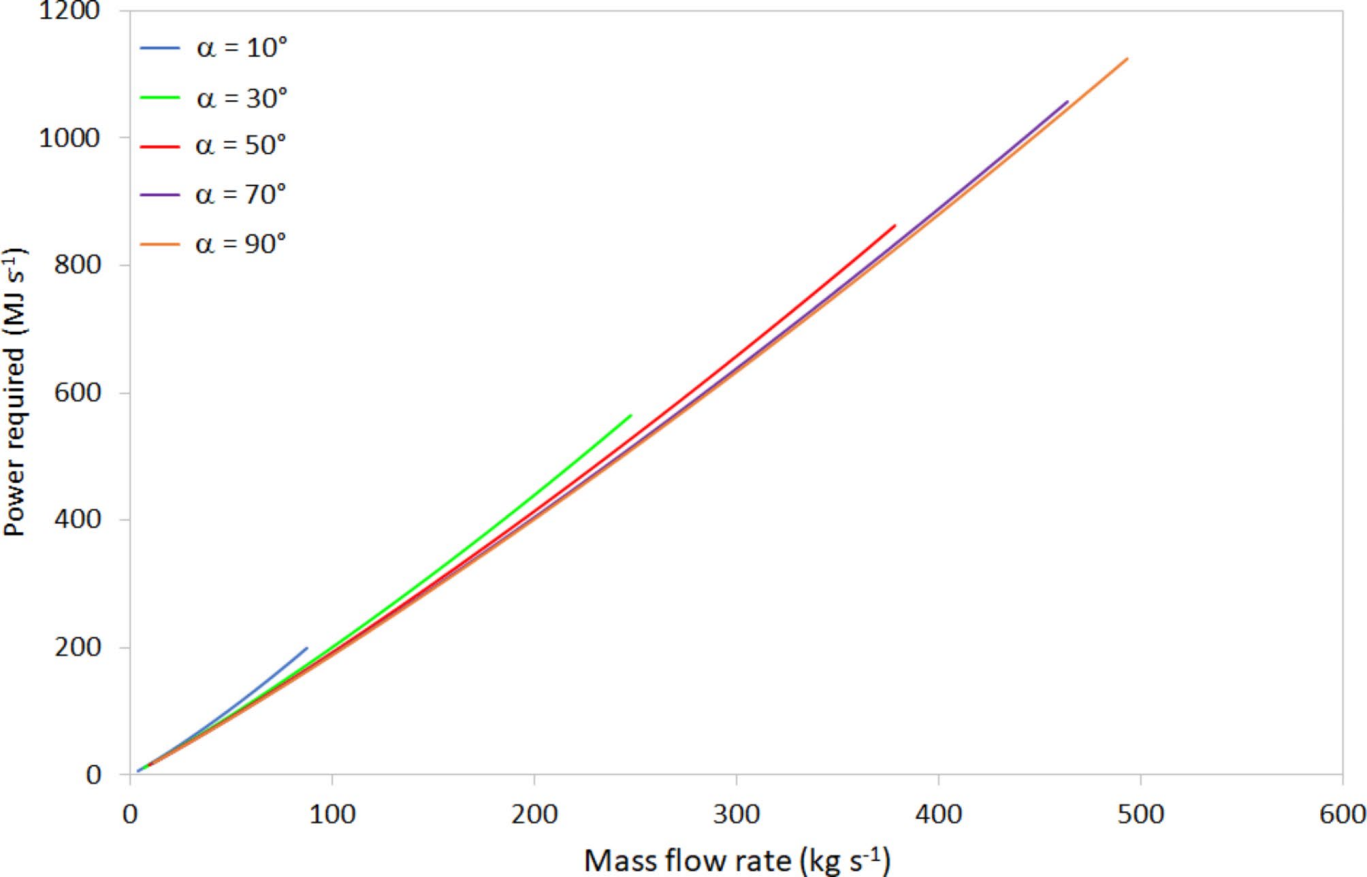
Relationship between mass flow rate of molten lunar regolith and the power required to maintain its temperature for conduit tilt angles, α, of 10°, 30°, 50°, 70°, and 90° from the horizontal (see Fig. 6 for schematic).

Using the values calculated in § 4.1, assuming a starting temperature of 100 K, Table 4 shows the specific energies required to heat regolith to melting and beyond – 1200 °C to 1600 °C. Also shown are the gravity-driven mass flow rate for MLR at that temperature assuming a conduit tilt angle of 10^o^ downwards (Eq. 11; Fig. 6), and the power required to maintain that mass flow rate. Table 5 shows the equivalent values for a conduit tilt angle of 90^o^ downwards. The relationships between mass flow rate and power required, for conduit tilt angles of 10^o^, 30^o^, 50^o^, 70^o^, and 90^o^ downwards, are shown in Fig. 8.

**Table 4:** Specific energy, mass flow rate, and power required to maintain the delivery of molten lunar regolith at a conduit tilt angle of 10^o^ downwards.

Temperature (°C)	Specific energy (MJ kg^-1^)	Mass flow rate (kg s^-1^)	Power required (MW)
1200	1.665	3.86	6.43
1300	1.819	11.7	21.3
1400	1.973	24.9	49.2
1500	2.127	38.5	87.9
1600	2.281	49.6	113.0

**Table 5:** Specific energy, mass flow rate, and power required to maintain the delivery of molten lunar regolith at a conduit tilt angle of 90^o^ downwards.

Temperature (°C)	Specific energy (MJ kg^-1^)	Mass flow rate (kg s^-1^)	Power required (MW)
1200	1.665	11.6	19.3
1300	1.819	56.7	103.1
1400	1.973	133.1	262.6
1500	2.127	211.4	449.6
1600	2.281	275.0	627.2

Consider the implications of the discrepancies between Eq. 2 and the measured temperature/viscosity data (Fig. 2), relating to the temperature-dependent viscosity of the MLR. At 1600 ^o^C, the calculated viscosity is $\:\eta\:=$ 0.11 Pa s (Eq. 2) yet the measured data shows $\:\eta\:=$ 0.05 Pa s. Considering the point of maximum deviation at a tilt angle of 90^o^ downwards, a viscosity of 0.05 Pa s would generate a mass flow rate of 493 kg s^-1^, compared to the 275 kg s^-1^ estimated for 0.11 Pa s viscosity. The higher mass flow rate would require 1.125 GW to maintain temperature and flow, compared to 0.627 GW for the lower mass flow rate (Table 5).

### The ingress of melt into porous structures

One possible use for molten regolith is as a sealant for porous structures, making them suitable for manufacturing sealed surfaces such as roads or runways. To achieve this, molten regolith could be poured on top of compacted, porous beds of regolith, whereupon the melt will infiltrate the pores between particles. The surface tension-driven ingress of a liquid into porous structure, whereby the cross-section of any conduit is not necessarily filled with liquid, is approximated by the Washburn equation, Eq. 12 ref^48^:

12 $$\:t=\frac{4\eta\:{L}^{2}}{\gamma\:d\text{cos}\theta\:}$$

where $\:\eta\:$ is the dynamic viscosity, $\:\gamma\:$ is the surface tension, $\:\theta\:$ is the contact angle between the liquid and the solid surface, and $\:t$ is the time required for the liquid to move distance $\:L$ along a conduit of diameter $\:d$.

As an example of the crucial influence of temperature and viscosity, consider the situation whereby MLR is being added to a porous structure consisting of interconnected cylindrical pores, to fill those pores. Assuming an average cavity diameter of 100 mm, a desired fill thickness of 500 mm, the contact angle is $\:\theta\:=$ 10^o^, and the liquid surface tension is $\:\gamma\:=$ 0.35 N m^-1^. At $\:T=$ 1200 °C the dynamic viscosity is $\:\eta\:=$ 3.48 Pa s, and the estimated time required to fill the structure is 1,011 s, i.e. approximately 17 min. In contrast, at $\:T=$ 1600 °C the dynamic viscosity is $\:\eta\:=$ 0.113 Pa s, and the estimated time required is just 33 s.

The ingress of MLR into porous solid structures of much lower temperature than the liquid would occur in such a way that the MLR would not retain a constant temperature. There would be substantial conductive heat losses, reducing the MLR viscosity, likely influencing its surface tension also. The MLR may even solidify before travelling very far if its initial temperature were insufficiently high. There will be an interplay between MLR cooling and the extent to which it could penetrate a porous structure. Theoretical analysis of this scenario would be beneficial in planning for any practical application.

## Conclusion

The results presented in this work are based on analysis of empirical data obtained via a technically demanding experiment performed using an instrument which requires a specialist operator. Though the numerical values used in this work are only a guide, they reveal useful insights relating to the design of high-temperature processes on the lunar surface. Conceiving a system for transporting and depositing molten regolith where the temperature is maintained close to a setpoint value will benefit from design optimisation, particularly for an extrusion-based 3D printing process. The molten regolith could lose energy via thermal radiation at hundreds of kW m^-2^, so minimising the surface area of exposed conduits and storage vessels maximizes energy efficiency. Construction material choices for any such network have great influence, with thermal conductivity and emissivity just two parameters that will affect the rate of energy loss from the system. All of these factors should be accounted for when constructing an energy balance for regolith transit systems. Further, small errors or uncertainties such as those associated with the empirical fit of measured viscosity data could lead to large variations in estimated mass flow rates. Analyses which consider the energy requirements for ISRU on the lunar surface should consider how much contingency to build into their plans.

Minimising the time required to perform unit operations by reducing the viscosity of molten regolith could yield power savings overall, supporting energy efficiency in an environment where energy sources are scarce. However, a balance must be sought between those viscosity-dependent parameters which dictate performance, particularly in the context of additive manufacturing^49^. For example, low viscosity fluids exhibit poor buildability yet good workability, whereas high viscosity liquids exhibit poor extrudability and printability. Too narrow a conduit diameter (e.g. 3D printing nozzle) will exhibit poor buildability, disrupting the bonding between layers due to longer printing times. In contrast, too wide a conduit diameter may also exhibit poor buildability in terms of filament deformation, perhaps due to shrinkage during cooling.

Parameters which require careful consideration – and experimental evaluation – include the temperature-dependent surface tension of the melt, which influences the wetting, flow, and handling behaviour. It should also be assessed to what extent the emissivity increases with increasing temperature, and whether molten regolith and solid regolith exhibit substantially different emissivities. The possibility of crystallization occurring during cooling once temperatures reach < 1250 °C should be investigated using X-ray diffraction. Other parameters ripe for investigation include how cooling rate determines which phases crystallize, whether the resulting solids are porous, glassy, or rock-like, and their mechanical strengths. Finally, it should be noted that there are many regolith compositions under consideration in this field of research, whose composition affects all of the physical properties discussed in this work.

## Electronic supplementary material

Below is the link to the electronic supplementary material.

Supplementary Material 1

## Data Availability

The datasets used and/or analysed during the current study are available from the corresponding author on reasonable request.
