# Supplementary material for: The viscosity and processing of molten lunar regolith
Source: Sci Rep. 2025 Jan 31;15:3938. doi: 10.1038/s41598-025-87761-7 (PMC11785779; doi:10.1038/s41598-025-87761-7)
Supplement: Supplementary file 1 — Supplementary Material 1 [file 41598_2025_87761_MOESM1_ESM.pdf]

The viscosity and processing of molten lunar regolith

James Bowen[1]*, Vibha Levin Prabhu[2], Sungwoo Lim[3], Mahesh Anand[1]
1. Faculty of Science, Technology, Engineering and Mathematics, The Open University, Walton Hall, Milton Keynes, MK7 6AA, UK
2. European Space Resources Innovation Centre, Luxembourg Institute of Science and Technology, L-4422, Luxembourg
3. Surrey Space Centre, University of Surrey, Guildford, Surrey, GU2 7XH, UK
* Corresponding author
Email: james.bowen@open.ac.uk
Telephone: + 44 (0) 1908 655 614

Supplementary Information

1. Droplet shape

With reference to **Figure 3** in the manuscript:

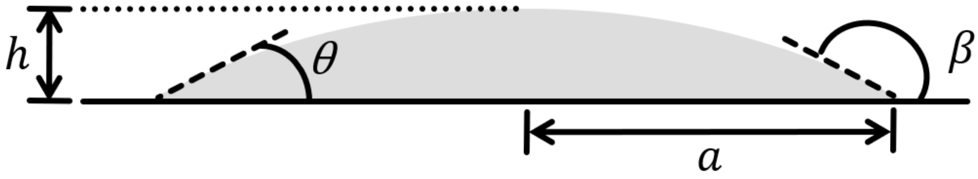

Figure 3. Droplet shape and associated parameters for molten regolith on a planar substrate.

The relationship between liquid density, ρ, droplet mass, m, and droplet volume, V:

$$V = \rho m \quad \text{(S1)}$$

The volume of a liquid droplet whose shape is approximated by a spherical cap:

$$V = \frac{\pi h}{6}(3a^2 + h^2) \quad \text{(S2)}$$

where a is the droplet radius and h is the droplet height at its centre.

The surface area, S, of a liquid droplet whose shape is approximated by a spherical cap:

$$S = \pi(a^2 + h^2) \quad \text{(S3)}$$

The relationship between the droplet radius, a, droplet height, h, and the radius of curvature of the equivalent sphere, R:

$$a = [h(2R - h)]^{0.5} \quad \text{(S4)}$$

The relationship between the droplet radius, a, and the radius of curvature of the equivalent sphere, R:

$$R = \frac{h}{1 - \sin\beta} \quad \text{(S5)}$$

where β is the angle between the three-phase line tangent and the surface, drawn outside of the droplet.

The relationship between the contact angle, θ, and the angle between the three-phase line tangent and the surface, β:

$$\theta = 180 - \beta \quad \text{(S6)}$$

For the situation described in this work there were six unknowns - β, a, h, R, S, and V - and there are six equations. The variable solved for was the droplet height, h.

2. Confirmation of JSC-1A melting using X-Ray Diffraction

Variable temperature X-ray diffraction was employed to verify that JSC-1A is molten in the temperature range investigated in this work, 1200-1600 °C. The instrument used was a Bruker D8 Advance Twin X-ray Diffractometer incorporating an Anton Paar HTK 2000N stage, using a resistively-heated tungsten strip as the substrate onto which JSC-1A was deposited, heated, and cooled. The spectra in **Figures S1-S3** show the diffraction pattern recorded sequentially at 25 °C, 1500 °C, and then 1200 °C respectively.

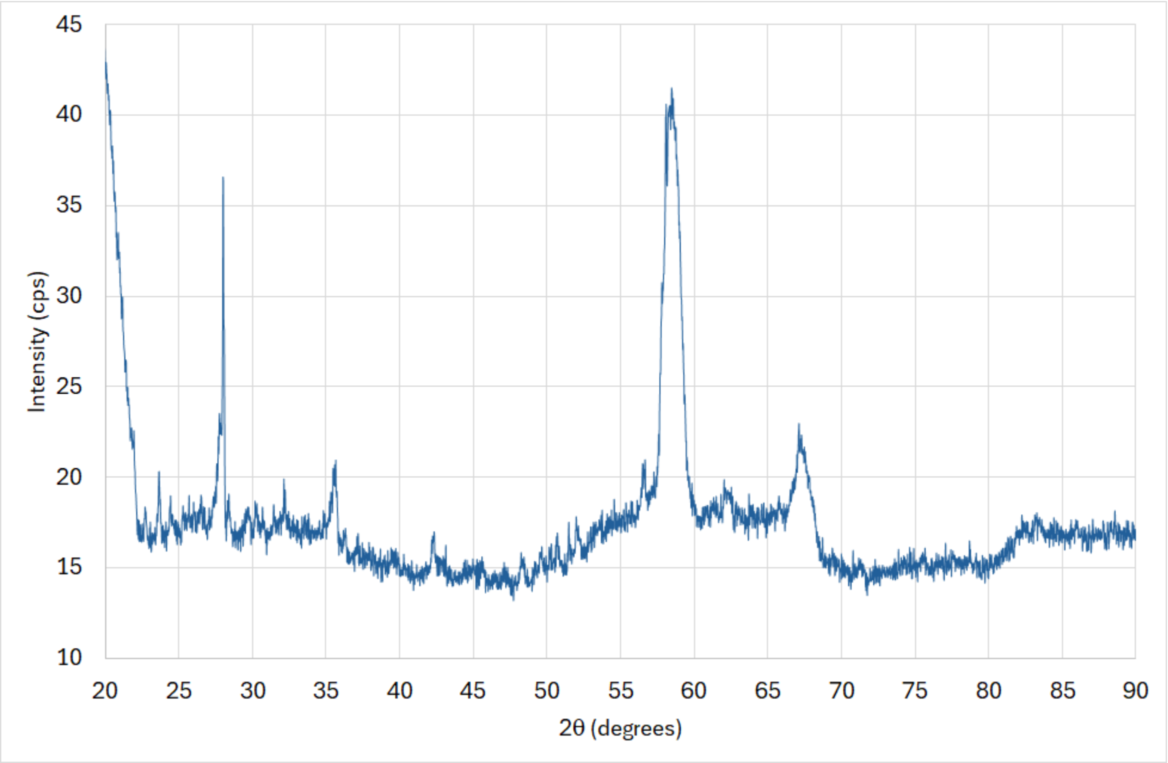

Figure S1. X-ray diffraction pattern for JSC-1A recorded at a temperature of 25 °C. The tungsten substrate yields peaks at 2θ = 32°, 40°, 52°, 58°, 67°, and 86°. All other peaks are from the solid JSC-1A.

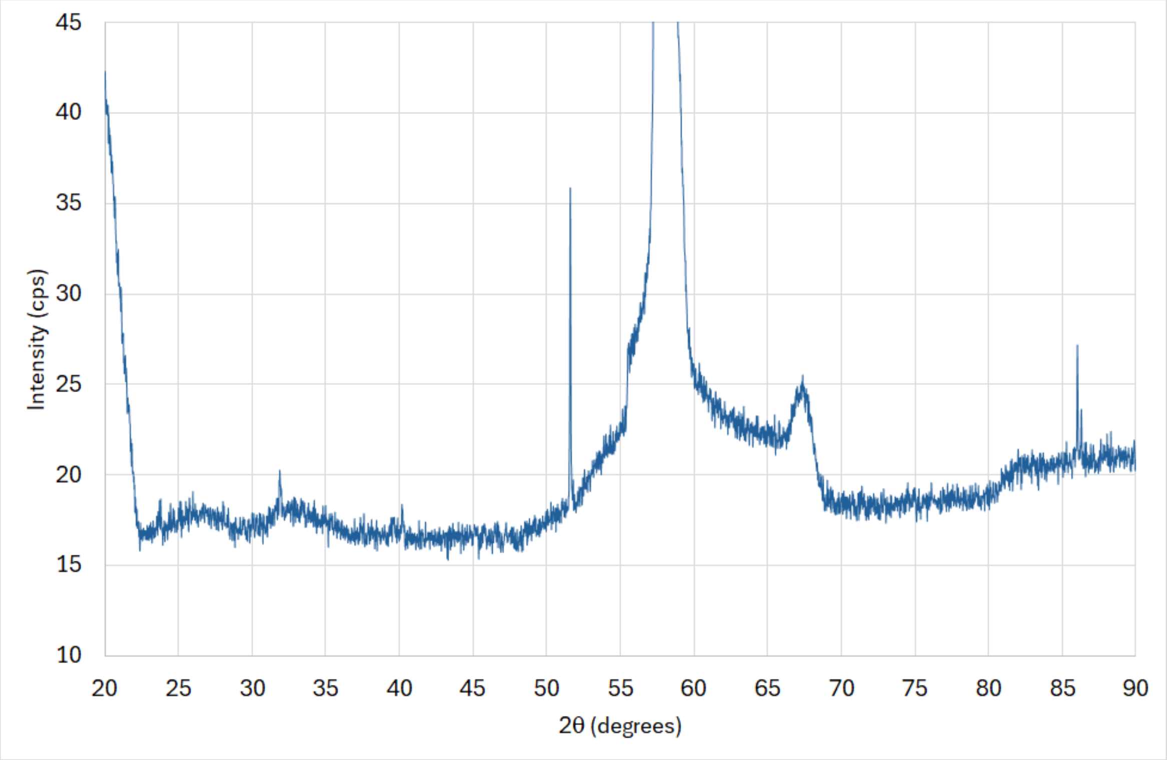

Figure S2. X-ray diffraction pattern for JSC-1A recorded at a temperature of 1500 °C. The tungsten substrate yields peaks at 2θ = 32°, 40°, 52°, 58°, 67°, and 86°. All peaks from the solid JSC-1A are absent, indicating that the solid powder has completely melted.

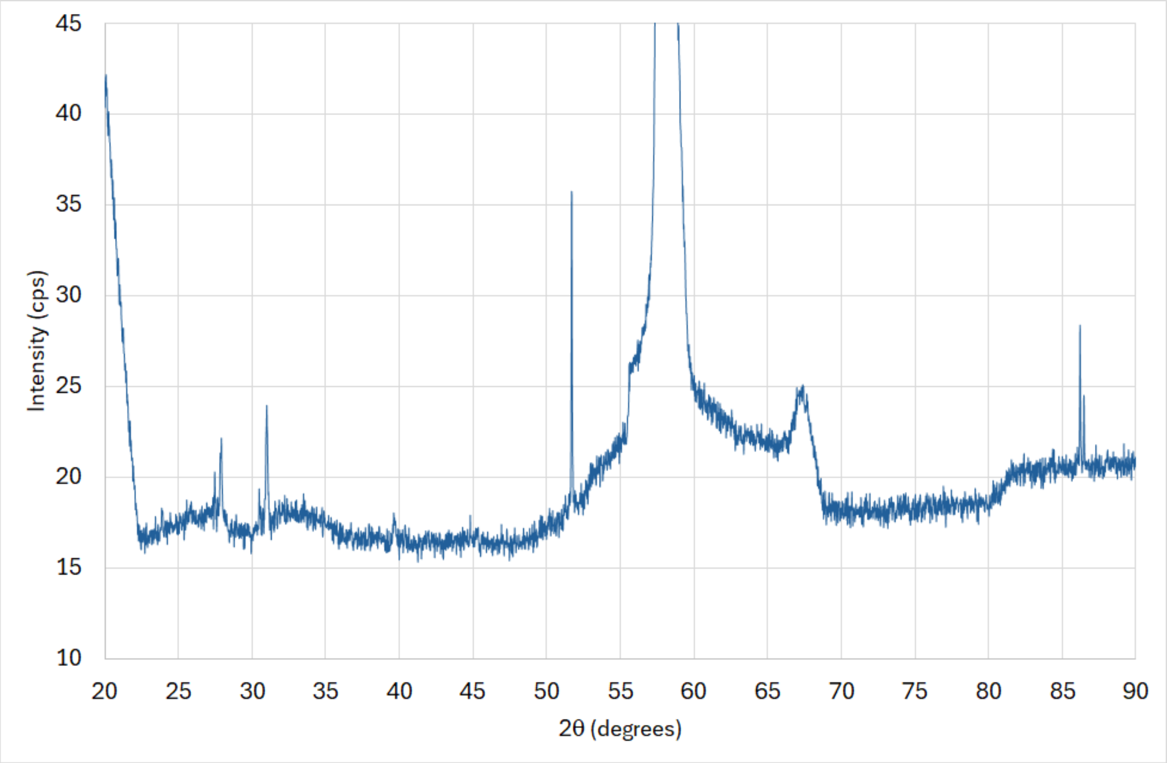

Figure S3. X-ray diffraction pattern for JSC-1A recorded at a temperature of 1200 °C. The tungsten substrate yields peaks at 2θ = 32°, 40°, 52°, 58°, 67°, and 86°. The appearance of a peak at 2θ = 28° suggests crystallites are beginning to form at this temperature, and hence this was the lowest temperature measured using furnace rheometry.
